# Development of a Contractile Cardiac Fiber From Pluripotent Stem Cell Derived Cardiomyocytes

**DOI:** 10.3389/fcvm.2018.00052

**Published:** 2018-06-11

**Authors:** Katrina J. Hansen, Michael A. Laflamme, Glenn R. Gaudette

**Affiliations:** ^1^Department of Biomedical Engineering, Worcester Polytechnic Institute, Worcester, MA, United States; ^2^Toronto General Hospital Research Institute, McEwen Centre for Regenerative Medicine, University Health Network, Toronto, ON, Canada

**Keywords:** pluripotent stem cell derived cardiomyocytes, high density mapping, contractile strain, fibrin microthreads, cardiovascular regeneration

## Abstract

Stem cell therapy has the potential to regenerate cardiac function after myocardial infarction. In this study, we sought to examine if fibrin microthread technology could be leveraged to develop a contractile fiber from human pluripotent stem cell derived cardiomyocytes (hPS-CM). hPS-CM seeded onto fibrin microthreads were able to adhere to the microthread and began to contract seven days after initial seeding. A digital speckle tracking algorithm was applied to high speed video data (>60 fps) to determine contraction behaviour including beat frequency, average and maximum contractile strain, and the principal angle of contraction of hPS-CM contracting on the microthreads over 21 days. At day 7, cells seeded on tissue culture plastic beat at 0.83 ± 0.25 beats/sec with an average contractile strain of 4.23±0.23%, which was significantly different from a beat frequency of 1.11 ± 0.45 beats/sec and an average contractile strain of 3.08±0.19% at day 21 (*n* = 18, *p* < 0.05). hPS-CM seeded on microthreads beat at 0.84 ± 0.15 beats/sec with an average contractile strain of 3.56±0.22%, which significantly increased to 1.03 ± 0.19 beats/sec and 4.47±0.29%, respectively, at 21 days (*n* = 18, *p* < 0.05). At day 7, 27% of the cells had a principle angle of contraction within 20 degrees of the microthread, whereas at day 21, 65% of hPS-CM were contracting within 20 degrees of the microthread (*n* = 17). Utilizing high speed calcium transient data (>300 fps) of Fluo-4AM loaded hPS-CM seeded microthreads, conduction velocities significantly increased from 3.69 ± 1.76 cm/s at day 7 to 24.26 ± 8.42 cm/s at day 21 (*n* = 5–6, *p* < 0.05). hPS-CM seeded microthreads exhibited positive expression for connexin 43, a gap junction protein, between cells. These data suggest that the fibrin microthread is a suitable scaffold for hPS-CM attachment and contraction. In addition, extended culture allows cells to contract in the direction of the thread, suggesting alignment of the cells in the microthread direction.

## Introduction

Cardiovascular disease (CVD) continues to be one of the leading causes of death worldwide (1). Myocardial infarction, a type of CVD, can develop into heart failure due to the inability for the heart to regenerate itself after a massive loss of contractile myocytes. Treatment for heart failure is limited with the only clinically acceptable method to regenerate contractile function being a heart transplant. However, due to limited donor availability and the potential for immune rejection, a heart transplant is not an ideal treatment option (2). Consequently, researchers have investigated other therapeutic options for heart failure, such as cellular therapy. Many studies have delivered hMSCs (human mesenchymal stem cells) to animal infarct models as well as human patients in clinical trials; however, there has only been minimal improvement in left ventricular function (2, 3). Studies of pluripotent stem cell derived cardiomyocytes have shown promise in small animal models to improve function via attenuated left ventricular remodeling by paracrine effects and improvements in neovascularization (4–7). More recent studies using larger animal models have delivered human pluripotent stem cell derived cardiomyocytes (hPS-CM) to guinea pig (8) and macaque monkey (9) models of infarction and have demonstrated the ability to electrically couple to the host myocardium and remuscularize portions of the ischemic tissue. To deliver the cells to the infarcted tissue, groups traditionally use an intramyocardial injections which suffers from low engraftment rates (<10%) (10). Many valuable cells are being lost in the delivery process and are never able to reach the intended ischemic tissue.

Cardiac tissue engineering has allowed for the creation of a large mass of muscle to be used in myocardial regeneration strategies (11, 12). Many of the scaffolds used in cardiac tissue engineering allow for directed orientation to align cardiomyocytes and improve contractility and electrical conduction (13–16). Scaffold free techniques using cell sheet technology have the added advantage of allowing tissue formations without the use of exogenous materials (17, 18). However, many of the structural advantages of transplanting hPS-CM in infarct models using a cardiac patch or cell sheet are lost due to the formation of collagen interfaces between the host and graft tissue which limit the ability for the graft cells to migrate into the host tissue (13, 19).

Previously, we have developed a fibrin microthread suture that can be used for efficient cell delivery directly to the myocardium (20). These fibrin microthreads have been shown to support hMSC and C2C12 myoblast attachment and survival as well as direct cellular alignment for myoblasts (21). Additionally, previous studies have indicated that fibrin is a suitable biomaterial to support hPS-CM attachment and contraction (13, 22). However, it is unknown whether fibrin microthreads are capable of supporting hPS-CM attachment and directing cell functionality. The aim of this study is to develop and characterize an hPS-CM seeded fibrin suture for future use as a delivery platform for cardiac repair. Here we implement strategies to seed hPS-CM onto fibrin microthreads and characterize how these cells function on the microthreads and understand how their contractile properties may change over time in culture.

## Materials and Methods

### Seeding Platform for hPS-CM Attachment to Fibrin Microthreads

Fibrin microthreads were produced as described previously (23). Briefly, fibrinogen (70 mg/ml; MP Biomedical) and thrombin (8 U/ml; Sigma) were coextruded using a blending tip connector in a 10 mM HEPES (Calbiochem) buffered bath (pH 7.4). A custom built extrusion system was used to extrude microthreads at a flow rate of 0.23 mL/min through 0.38 mm polyethylene tubing. After extrusion, the microthreads were allowed to polymerize before being removed and air dried.

**Figure 1:**
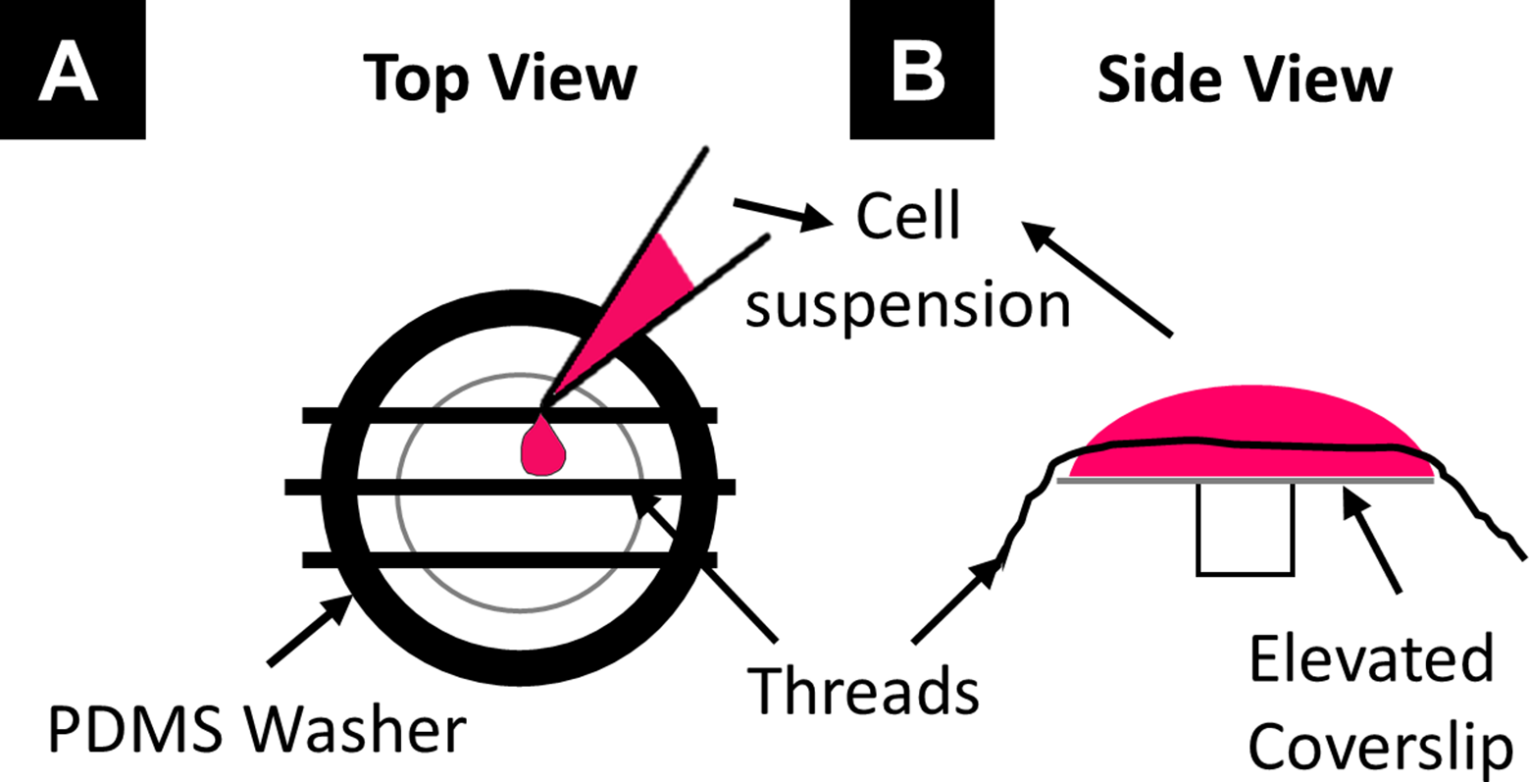
Seeding schematic to seed hPS-CM onto fibrin microthreads. Individual fibrin microthreads are glued to a PDMS washer **(A)** and then placed over an elevated 12mm coverslip **(B)**. A 150µL suspension of cells is pipetted onto the coverslip with the threads for cell seeding.

hPS-CM were generated using a previously described directed differentiation protocol using activin-A and bone morphogenetic protein-4 (4, 24). Cells were cryopreserved as previously reported (25), and all cell preparations contained >70% cardiac troponin T + cardiomyocytes by flow cytometry. To facilitate hPS-CM seeding individual fibrin microthreads were used and adhered in groups of three to custom polydimethylsiloxane (PDMS) washers (inner diameter: 1.2 cm) using medical grade silicone adhesive (Figure 1A). PDMS constructs with microthreads were placed in a six well plate for ethylene oxide (EtO) sterilization. Constructs were allowed to de-gas for 24 h after EtO sterilization. Immediately before seeding, microthreads were allowed to rehydrate for 20 min in dPBS. Thread constructs were then placed over an elevated 12 mm glass coverslip and coated with ECM protein (fibronectin or collagen IV, 10 µg/ml) for 2 h. For hPS-CM seeding, constructs were moved to new 12 mm glass coverslip platforms and a 150 µL cell suspension (1.33 × 10^6^ cells/ml) in aprotinin (50 µg/ml) supplemented RPMI-B27 medium was added to the coverslip (Figure 1B). Control TCP plates were coated with 0.1% gelatin, collagen IV, or fibronectin and were seeded with hPS-CM at a density of 150,000 cells/cm^2^. Cells were allowed to attach for 18 h in an incubator at 37°C, after which cell seeded microthreads were moved to a six well plate with fresh RPMI-B27 medium supplemented with aprotinin, medium was changed every 2–3 days.

### Effect of ECM Surface Coatings on hPS-CM Attachment

To examine the effect of different surface coatings on hPS-CM attachment to fibrin microthreads, ECM proteins collagen IV and fibronectin were used. After the threads were hydrated and moved to an elevated 12 mm coverslip, threads were coated with ECM proteins for 2 h after which constructs were moved to another elevated 12 mm coverslip for final cell seeding. Cells were seeded as described previously. 48 h after seeding, constructs were moved to new wells with fresh medium for continued culture, or microthreads were prepared for a CyQuant assay. All threads from one construct were cut and placed in an eppendorf tube containing 1 ml of dPBS and the CyQuant assay was run according to the manufactures specifications.

### Viability of hPS-CM Attachment on Fibrin Microthreads

To characterize cell viability a LIVE/DEAD assay (Life Technologies) was conducted, according to manufactures recommendations, 48 h after hPS-CM constructs were seeded. Briefly, medium was aspirated and replaced with 1 ml of sterile RPMI containing a 4 µM ethidium homodimer-1 and 2 µM calcein-AM working solution and plates were incubated at 37C for 15 min. The RPMI solution was aspirated and replaced with a 4 µM ethidium homodimer-1 and 2 µM calcein-AM working solution with Hoechst 33342 (0.5ug/mL; Life Technologies) and plates were incubated for an additional 15 min at 37C. Calcein-AM (green Ex/em 495 nm/515 nm) is retained within the cytoplasm of living cells, while ethidium homodimer-1 (red, Ex/em 495 nm/635 nm) enters dead cells and binds nuclear DNA, but is excluded from living cells with intact plasma membrane activity. Microthreads were imaged using a Leica DMIL inverted microscope.

### Capturing Contraction of hPS-CM Seeded Microthreads

To record the contraction produced by seeded sutures a LEICA DMIL inverted microscope with a Fastec HiSpec4 camera mounted to record video at high magnifications. Video was recorded at 60 frames per second for 25 s at a magnification level of 200x at 4 different locations along the length of the thread. To determine the time course of contraction, microthreads were recorded at days 7, 14, and 21 post seeding. Collagen IV, fibronectin, and fibrin only hPS-CM seeded sutures were analyzed. Parallel control experiments were run with hPS-CM plated on fibronectin (10 µg/ml) and collagen IV (10 µg/ml) coated TCP at a density of 150,000 cells/cm^2^. The data was analyzed to calculate average contractile strain, maximum contractile strain, and beat frequency using high density mapping (HDM), a speckle tracking algorithm, as previously described (26). For contractility alignment, the values of the principal angle were calculated for zero to peak of E2, for each contraction cycle, and were compared to the angle of the microthread and the difference between the two was determined. Aligned cells were defined as those with an angle differences between +/-0–20 degrees. The percent of cells contracting in alignment with the microthread was reported for days 7, 14, and 21.

### Conduction Velocity Measurements

To measure conduction velocity hPS-CM seeded sutures were loaded with Fluo-4 AM (5 µM dissolved in Pluronic F-127 (20% solution in DMSO); Invitrogen) and recorded at days 7, 14, and 21 using a previously defined system (26), within 2 h of dye addition. Briefly, a Zeiss AcioObserver.A1 inverted microscope with a Hamamatsu Orca Flash 4.0sCMOS camera was used to obtain fluorescent videos at greater than 70 frames per second. Calcium transient analysis of the individual frames was applied to 2–3 regions along the thread using a custom MATLAB code that determined the average fluorescence intensity change with respect to baseline intensity and was reported as ΔF/F_0_. Calcium transients for each region were then plotted together and the frame difference between the start frames of each calcium transient cycle for the different regions was recorded and the time delay was calculated. Next, the distance between the center points of each region was calculated using ImageJ. Conduction velocity was then calculated by dividing the distance between the regions by the time delay between the initiations of the calcium transients and is reported in cm/s.

### Immunocytochemistry

At day 1, 4, 7, 14, and 21 hPS-CM seeded microthreads were fixed in 4% paraformaldehyde for 10 min, rinsed with PBS and blocked in 5% goat serum or 1.5% rabbit serum in PBS for 45 min. Primary antibodies were applied at a dilution of 1:100 (mouse-anti-alpha actinin, Abcam; rabbit-anti-connexin-43, Cell Signaling Technologies) at 4°C overnight. Cells were incubated for an hour in the appropriate secondary antibodies (AF488 anti-mouse 1:400, AF568 anti-rabbit 1:400, Invitrogen). Cells were counterstained using Hoechst 33342 (0.5ug/mL, Life Technologies) for 5 min. Images were obtained using a Leica laser scanning confocal microscope.

### Alpha-Actinin Fiber Alignment

Confocal images of alpha-actinin stained hPS-CM seed on microthreads and TCP were uploaded to ImageJ (NIH) for actinin fiber alignment using OrientationJ. Briefly, each image was split into its green channel and each section of fibers were outlined and analyzed. Output angle data for each image was averaged and compared to the angle of the thread and reported as the difference between the thread angle and the average fiber angle.

### Statistical Analysis

Statistical analyses were performed using GraphPad Prism 6 (GraphPad Software, Inc.). Comparisons between seeding conditions, alignment data, and conduction velocities were analyzed using a one-way ANOVA, with a post-hoc Tukey test for multiple comparisons. Comparisons for contractile parameters of hPS-CM seeded on different surface coatings (threads and TCP) between time points were done using a two-way ANOVA with a Tukey post-hoc analysis for multiple comparisons. Comparisons were done including all data from threads and TCP, and separately with data from just plates groups and just threads groups to elucidate differences washed out when all groups across time and culture substrates were compared. A Pearson linear correlation coefficient was calculated in excel using the PEARSON function to assess the strength of correlation between contractile alignment and average contractile strain. Cell attachment number, average and maximum contractile strain, actinin alignment, and conduction velocity are reported as mean ±SEM, frequency data is reported as mean ±SD. Significance was considered at *p* < 0.05.

## Results

### Collagen IV Coating Improves hPS-CM Attachment to Fibrin Microthreads

**Figure 2:**
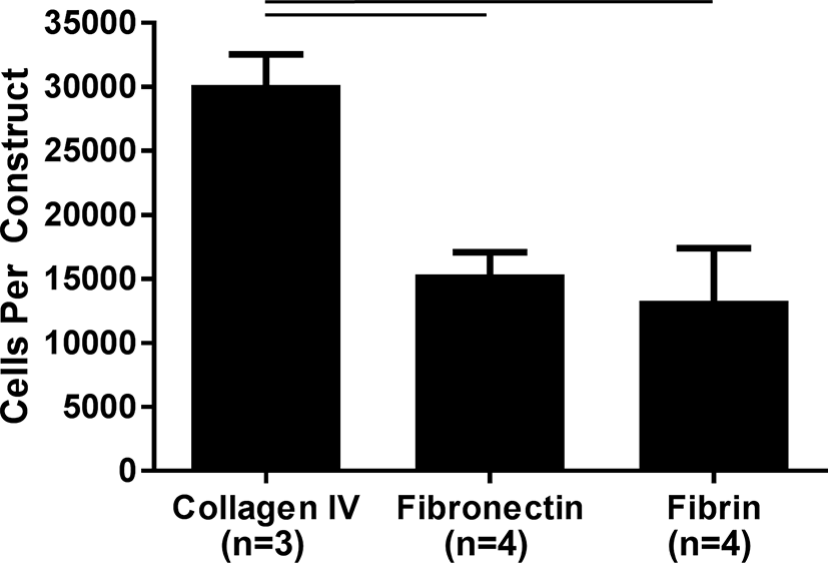
Collagen IV ECM coating enhances hPS-CM attachment to fibrin microthreads. Fibrin microthreads coated with collagen IV improved hPS-CM attachment over fibronectin coated or non-coated fibrin microthreads. Mean±SEM. n=3-4, p<0.05.

Fibronectin and collagen IV ECM protein coatings were evaluated against fibrin only to determine how ECM protein coating would affect hPS-CM attachment. Immunohistochemistry confirmed that both fibronectin and collagen IV proteins coatings resulted in positive expression of fibronectin and collagen IV on the surface of the microthread. Cell attachment was measured and quantified 2 days post seeding using a CyQuant DNA assay. hPS-CM attachment was observed for all conditions, with significantly higher cells attached on the collagen IV coated microthreads (*n* = 3–4, *p* < 0.05, Figure 2). Increases in cell attachment and using different protein coatings did not appear to affect cell viability as indicated qualitatively by a LIVE/DEAD stain (Figure S1).

### hPS-CM Exhibit Opposite Temporal Trends in Contractile and Maximum Strains When Cultured on TCP and Fibrin Microthreads

**Figure 3:**
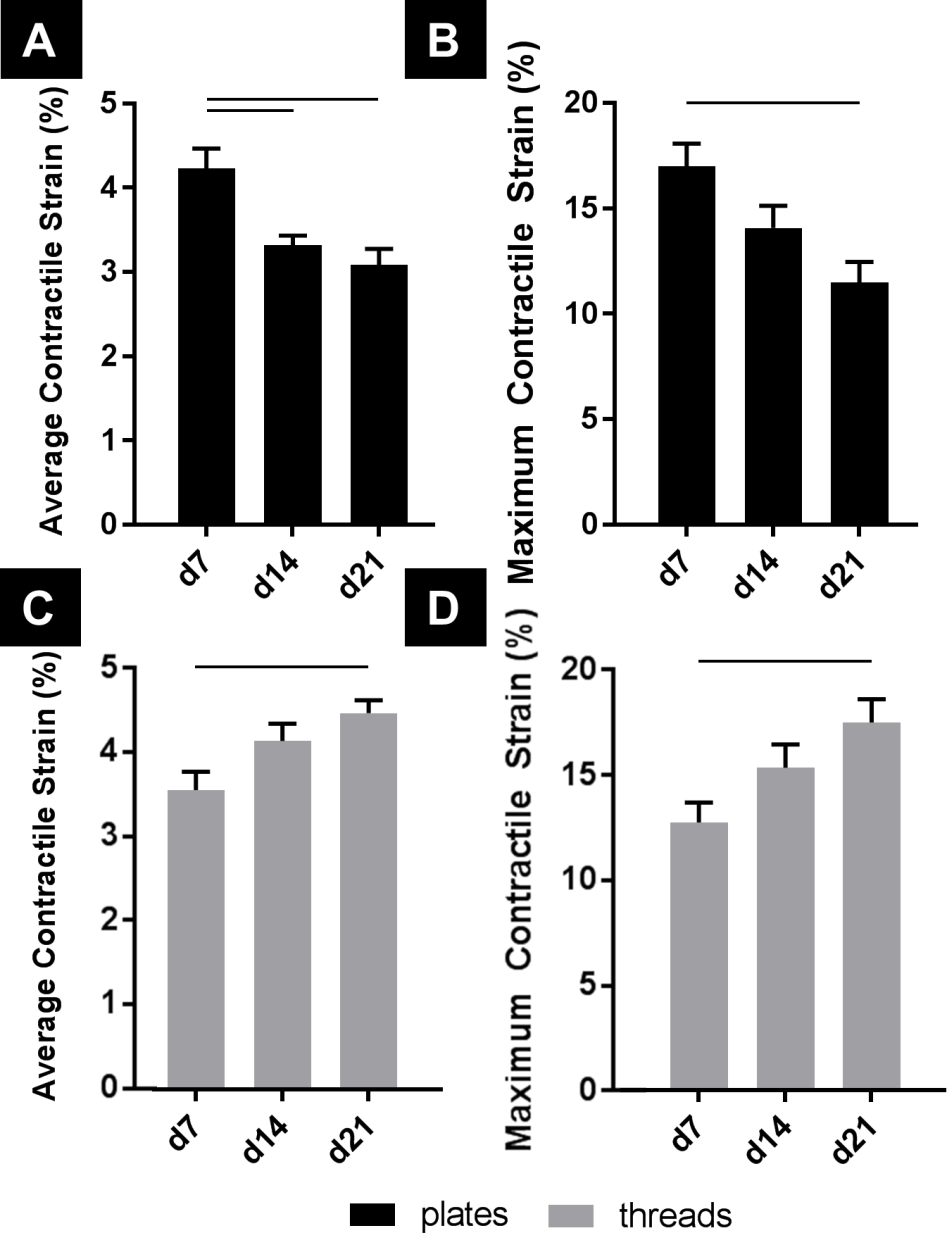
hPS-CM exhibit opposite trends in contractile and maximum strains when cultured on TCP and fibrin microthreads over 21 days. hPS-CM exhibit a significant decrease in contractile **(A)** and maximum strains **(B)** when cultured on TCP over 21 days. Conversely, hPS-CM cultured on fibrin microthreads exhibit an increase in contractile **(C)** and maximum strains **(D)** over 21 days. Mean±SEM for contractile and maximum strain, n=18, —p<0.05.

To examine contractile behavior, average contractile strain and maximum contractile strains were evaluated from hPS-CM seeded TCP (0.1% gelatin, collagen IV, and fibronectin coated) and microthreads (fibrin, fibronectin and collagen IV coated) at days 7, 14, and 21 post seeding. On TCP both average contractile strains and maximum contractile strains followed similar trends such that cells cultured on TCP produced decreased strains over 21 days. Cells on TCP exhibit a significant decrease in average contractile strain over time, beginning at 4.23±0.23% on day 7 and ending at 3.08±0.19% on day 21 (Figure 3A, *n* = 18, *p* < 0.05). Maximum contractile strain followed a similar trend; cells on TCP had a maximum contractile strain of 17.01±1.07% on day 7, which significantly decreased to 11.48±0.99% on day 21 (Figure 3B, *n* = 18, *p* < 0.05). hPS-CM seeded on microthreads increased average contractile strain and maximum contractile strain over 21 days. At day 7, hPS-CM seeded on microthreads begin at an average contractile strain of 3.56±0.22% and increased average contractile strain to 4.47±0.29% on day 21 (Figure 3C, *n* = 18, *p* < 0.05). In terms of maximum contractile strain, hPS-CM seeded on microthreads produced a maximum contractile strain on day 7 of 12.70±0.94% which significantly increased to a maximum contractile strain of 17.44±1.09% on day 21 (Figure 3D, *n* = 18, *p* < 0.05).

**Figure 4:**
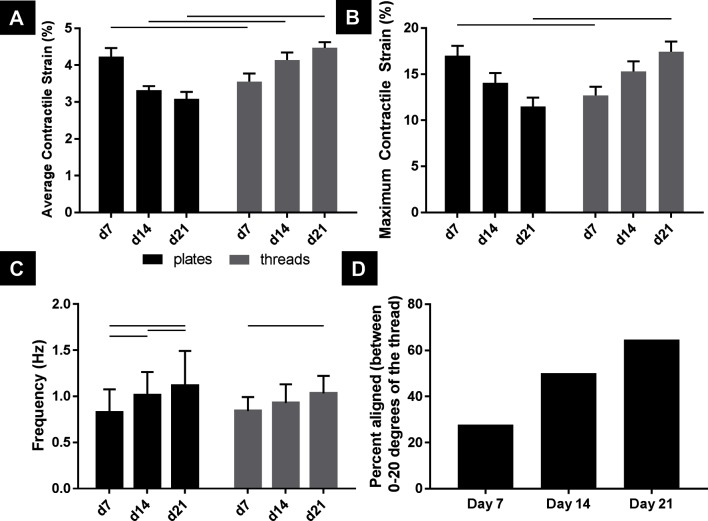
hPS-CM exhibit significantly higher contractile and maximum strains when cultured on fibrin microthread compared to TCP by 21 days with increases in frequency and alignment over time. hPS-CM cultured on fibrin microthreads **(A)** exhibit significantly different contractile strains, at all-time points, and maximum strains, at days 7 and 21, than cells cultured on TCP **(B)**. hPS-CM significantly increased beat frequency between day 7 and day 21 for both culture conditions **(C)**. Over 21 days in culture a higher percentage of hPS-CM contract in the same direction (within 20 degrees) as the microthread **(D)**. Mean±SEM for average contractile and maximum strain, Mean±SD for frequency, n=18 [n=17 for day 21 **(D)**],—p<0.05.

Looking between culture substrate and over time, cells cultured on TCP and microthreads exhibited significantly different average contractile strains on days 7, 14, and 21, and significantly different maximum contractile strains on days 7 and 21 (Figure 4A,B, *n* = 18, *p* < 0.05). Both average contractile strain and maximum contractile strain were higher at day 21 for hPS-CM cultured on fibrin microthreads than the strains produced by hPS-CM cultured on TCP at day 7. Average contractile strain and maximum contractile strain followed similar trends between coating groups, on TCP and on microthreads over 21 days (Figure S2).

### Contractile Frequency Increases Over 21 Days for hPS-CM Cultured on TCP and Microthreads

Beat frequency was examined to determine if there were any changes in beat rate for hPS-CM seeded on microthreads or TCP over 21 days (Figure 4C). Frequency significantly increased from day 7 to day 21 for both TCP and microthread groups; from 0.83 ± 0.25 Hz to 1.11 ± 0.45 Hz for the TCP group and from 0.84 ± 0.15 Hz to 1.03 ± 0.19 Hz for the microthread group (*n* = 18, *p* < 0.05). No differences were found between TCP and microthread groups for any time points. Frequency was found to increase over time for all surface coatings, on microthreads and TCP, with the exception for the gelatin coated group which decreased between day 14 and 21 (Figure S2).

### hPS-CM contraction direction becomes more aligned with the thread over 21 days in culture

 hPS-CM seeded on non-coated, collagen IV and fibronectin coated fibrin microthreads were used to examine changes in cellular alignment to the fibrin microthreads in terms of direction of principal contraction (*n* = 17–18, Figure 4D). Values reported are the percentage of cells that contracted within 0–20 degrees of the direction of the fibrin microthread. At day 7 only 27% of cells contracted within 20 degrees of the direction the fibrin microthread, this increased to 65% by day 21. Additionally, hPS-CM seeded on TCP did not exhibit the same trend over 21 days in culture as cells had wide ranges of principal contraction angles at all-time points indicating that they remained unaligned over 21 days (data not shown).

When contractile alignment was plotted together with changes in average contractile strain over 21 days there appeared to be a linear relationship between contractile alignment and average contractile strain for hPS-CM seeded on sutures (Figure S3). A Pearson linear correlation coefficient of 0.99 between variables was found indicating a strong correlation between increases in contractile alignment and average contractile strain. For hPS-CM cultured on TCP average contractile strain decreased over 21 days with no major change in alignment as indicated by the flat orange trendline in Figure S3 and a Pearson linear correlation coefficient of −0.20.

### hPS-CM Seeded on Microthreads Increase Conduction Velocity Over 21 Days in Culture

**Figure 5:**
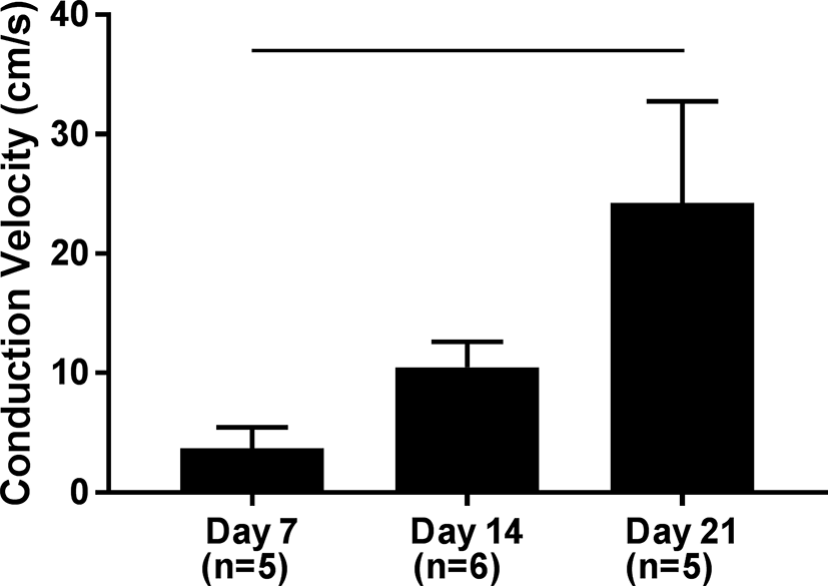
Conduction velocity of hPS-CM seeded sutures. High speed videos of Fluo-4 loaded hPS-CM seeded on fibrin microthreads were analyzed for calcium transient intensity over different regions along the microthread. Conduction velocities were found to significantly increase over 21 days in culture. Mean±SEM, n=5-6, -p<0.05.

Using the system previously developed (26) we examined calcium transients in terms of conduction velocity of hPS-CM seeded on fibrin microthreads. High speed videos of hPS-CM seeded on microthreads loaded with Fluo-4 AM dye were analyzed for calcium transient intensity over 2–3 regions along the microthread to determine conduction velocity at days 7, 14, and 21. Conduction velocities significantly increased from 3.69 ± 1.76 cm/s at day 7 to 24.26 ± 8.42 cm/s at day 21 (Figure 5, *n* = 5–6, *p* < 0.05).

### hPS-CM Seeded Microthreads Express Alpha-Actinin and Connexin 43

**Figure 6:**
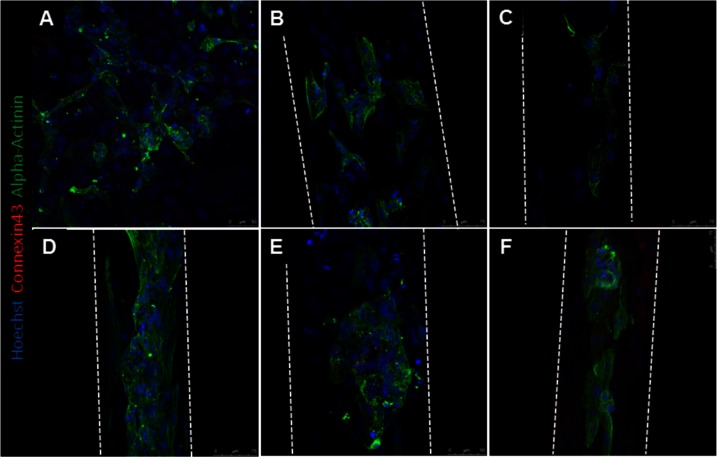
hPS-CM seeded onto fibrin microthreads express alpha-actinin and connexin 43. hPS-CM seeded onto tissue culture plastic **(A)** and collagen IV coated fibrin microthreads express alpha-actinin and connexin 43 proteins at days 1 **(B)**, 4 **(C)**, 7 **(D)**, 14 **(E)**, and 21 **(F)** in culture. Cells seeded on tissue culture plastic exhibit no dominant alignment. Cells at later time points are more aligned along the length of the fibrin microthread.

**Figure 7:**
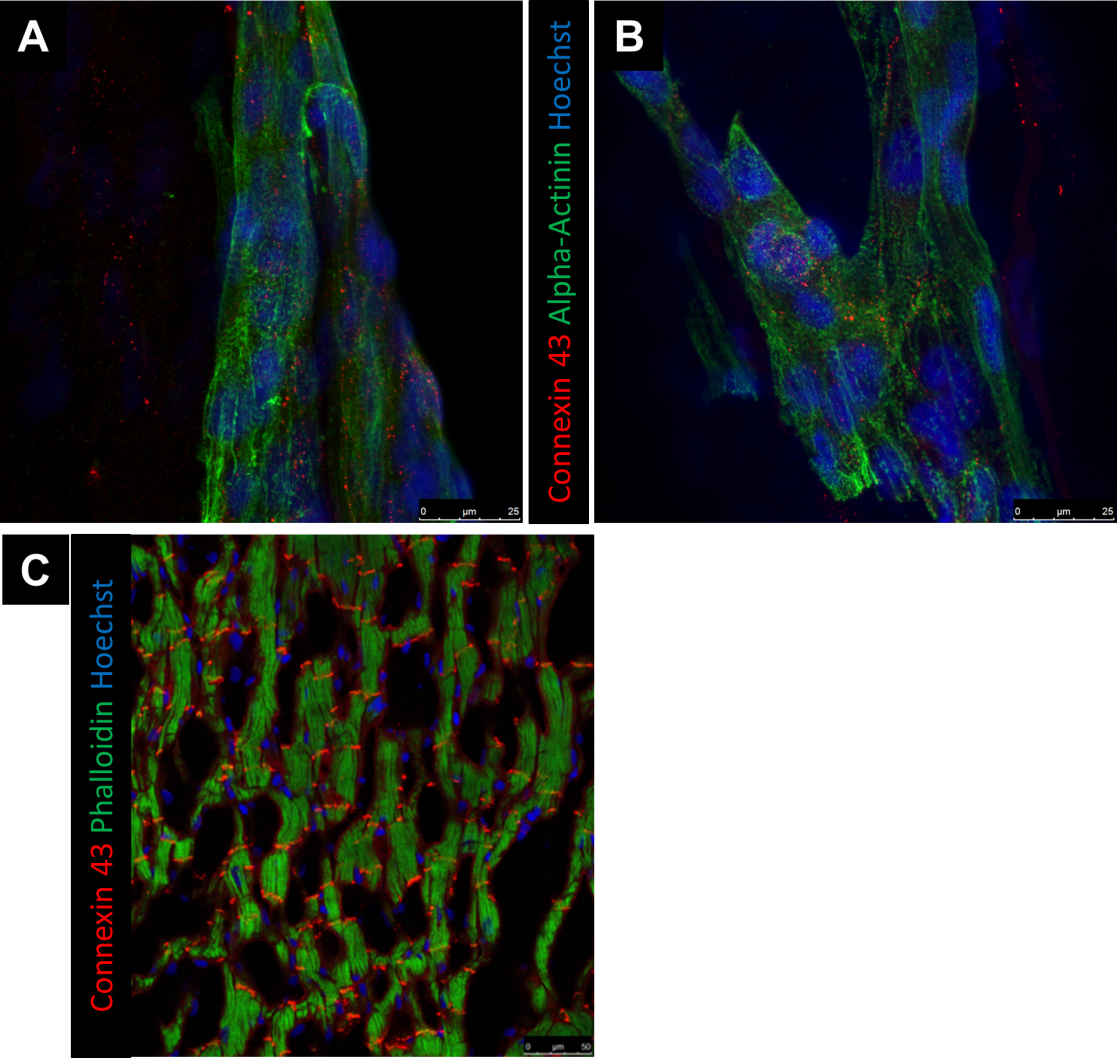
Representative images of Connexin 43 at day 21. Connexin 43 expression of hPS-CM were seeded onto fibrin microthreads at day 21 **(A,B)** does not mimic the end to end localization of Connexin 43 expression expected for ventricular myocytes **(C)**.

Alpha-actinin and connexin 43 staining were utilized to determine hPS-CM morphology up to 21 days in culture. hPS-CM were readily attached to microthreads at all-time points and exhibited positive alpha-actinin and connexin 43 staining (Figure 6). Cells were less elongated at the earlier time points (1 and 4 days). By day 7 cells had begun to elongate along the direction of the microthread and exhibited more organized sarcomere structure. Quantifying alignment demonstrated that hPS-CM at day 7 were aligned within 13.7 ± 6.2 degrees to the thread, compared to 11.7 ± 5.8 and 8.0 ± 1.1 degrees on days 14 and 21, respectively (*n* = 4–27 images, NS, Figure S4). Cells on TCP exhibited no dominate fiber alignment as indicated by a range of fiber angles from −86.6 to 86.4 degrees (*n* = 43 cell regions, data not shown) Cells demonstrated positive connexin 43 staining; however, at all-time points the staining was diffuse through the cells with no evidence of organization and polarization to the intercalated disks (Figure 7).

## Discussion

The goals of this study were to define the seeding conditions and time to obtain a contractile hPS-CM seeded microthread and characterize the cells contractile behavior when seeded on the microthreads. By obtaining a contractile cardiomyocyte microthread the microthreads can be attached to a suture needle in such a way that allows for targeted cell delivery to cardiac tissue. This study did not examine cell delivery to cardiac tissue via the microthreads, however previous work in our lab has suggested the feasibility of efficient and directed microthread based cell delivery (20). This cell delivery platform could provide an improved cell delivery method for regenerative medicine strategies seeking to treat heart failure. In this work, different ECM surface coatings were used to improve cell attachment. Fibronectin and collagen IV were chosen due to their presence in cardiac basement membrane. Additionally, other studies seeding hPS-CM on various substrates have demonstrated successful hPS-CM attachment using fibronectin and collagen IV coatings (27, 28). hPS-CM attached with higher quantities to collagen IV coated microthreads two days after seeding. All conditions did allow hPS-CM attachment, suggesting that hPS-CM preferentially attached to collagen IV coated microthreads, but a protein coating is not necessary to obtain hPS-CM attachment on fibrin microthreads. Qualitatively, protein coatings did not affect cell viability as the majority of cells attached were found to be viable for all conditions.

In regards to contractility, hPS-CM seeded on microthreads began to contract within 7 days after seeding and cell contraction was observed for all conditions. Beat frequency increased over 21 days for hPS-CM seeded on microthreads and on TCP to above 1 Hz, a similar finding to other groups (29, 30). Average contractile strain and maximum contractile strain was found to increase over 21 days for hPS-CM seeded on microthreads. These values, at all-time points, were significantly different than the average contractile strain and maximum contractile strain produced by hPS-CM on TCP, which were found to decrease over 21 days. Maximum contractile strains produced by hPS-CM on the microthreads near the 19% strain that is produced by adult human myocardium (31). Other tissue engineered constructs, such as the engineered heart tissue created by Eng et al. have demonstrated strains upwards of 17%, although these tissues needed to be electrically stimulated to produce higher strains where the unstimulated controls only produced strains of 10% (30).

Cardiomyocytes exist in an environment that requires them to be highly aligned and organized to produce efficient contractions. Previous studies have suggested the ability for fibrin microthreads to direct cell orientation along the microthread (21). In order to investigate the relationship of alignment with strain changes over time we sought to quantify cell alignment to determine the effect cell alignment may have had on changes in strain. Within a given layer of healthy myocardium, cardiomyocytes are aligned within 13 degrees of each other (32). The same would be expected of hPS derived cardiomyocytes for efficient contraction of the heart.

We demonstrated that over 21 days in culture the direction of cell contraction was more aligned along thread. Additionally, alignment was confirmed by immunohistochemical stains as cells aligned more closely to the thread over 21 days with a final alignment within 8 degrees of the thread. Studies in two and three dimensions have demonstrated the importance of cardiomyocyte alignment on improving contractility compared to unaligned controls (15, 33). As average contractile strains and maximum contractile strains increased over 21 days for hPS-CM seeded on microthreads, this would suggest that these cells are able to produce higher strains because they are contracting in a direction more aligned with the microthreads. No principal contraction alignment was found for cells cultured on TCP, which may explain the decrease in strains produced by these cells over time. A study by Khan et al. demonstrated that hPS-CM cultured on a polylactide-co-glycolide nanofiber scaffold improved alignment and function compared to hPS-CM cultured on tissue culture plastic. These results supports the findings here where hPS-CM cultured on a fibrin microthread scaffold also influenced and improved cell alignment and function (34).

Conduction velocity in ventricular myocardial tissue is approximately 50cm/s (35, 36). Here, using calcium transient analysis we demonstrated conduction velocities that began at 3.7 cm/s on day 7, increasing by day 21 to a mean of 24.2 cm/s with some microthreads demonstrating conduction velocities reaching 46.3 cm/s. Not only do these conduction velocities near that of human myocardium, but increasing conduction velocities over time would suggest that this increase may be due to the higher alignment of hPS-CM over time. Other studies have indicated a similar trend in conduction velocities where aligned cultures of cardiomyocytes demonstrated a 1.6 increase in conduction velocity (37).

Alignment may not be the only contribution to changes in strain over time; substrate stiffness may also play a role in how much strain the cells can produce. While alignment may be driving an increase in contractile strain it is possible that substrate stiffness plays a role. Hydrated fibrin microthreads have a modulus value around 2.2 MPa (21) whereas tissue culture plastic has a modulus value on the gigapascal range (38) which is significantly stiffer than human myocardial tissue, which can range in stiffness from 20 to 500kPa (39). It has been demonstrated that increased cell functionality may be a function of both substrate stiffness and alignment (40–43). McDevitt et al. demonstrated that cardiomyocytes patterned on glass coverslips lost their alignment over 10 days in culture suggesting that the stiff glass substrate is unfavorable to cardiomyocytes even though they had originally been patterned in an aligned manner (42). Ribeiro et al. used matrigel micropatterns on physiologically relevant degrees of stiffness to constrain hPS-CMs into rectangular shapes that follow a physiological 7:1 aspect ratio (33). These patterned hPS-CM were capable of producing higher contractile forces and improved myofibril alignment. The studies by McDevitt and Ribeiro were done on glass coverslips, however cardiomyocytes exist in a 3D environment and thus it is important to consider the effects of alignment and stiffness in an environment that better mimics myocardium. Chrobak et al. examined cardiomyocyte function using an unaligned fibrin gel with a modulus of approximately 0.25 kPa (44). They demonstrated contractile strains decreased over time suggesting that in a soft unaligned environment cardiomyocytes are unable to increase strains over time. Black et al. demonstrated that an aligned cardiomyocyte populated fibrin gel was capable of increased twitch forces at 2 weeks in culture over unaligned controls (45). These results, in conjunction with the data presented here, suggest that neither an unaligned soft substrate or an aligned stiff substrate are enough to improve cardiomyocyte function, but that a soft substrate that promotes alignment may be ideal for cardiomyocyte function.

When hPS-CM were seeded onto collagen IV coated tissue TCP, cells retained their contractile nature, but lacked a rod like phenotype with the organized sarcomere structure expected of mature cardiomyocyte. Connexin 43 is a protein found in cardiac connexons, which make up gap junctions that connect cardiomyocytes. To facilitate cardiac conduction, gap junctions are organized end-to-end at the intercalated disks within cardiac tissue. Qualitatively, no organizational differences in connexin 43 staining was found for hPS-CM seeded on TCP or fibrin microthreads at any time points. However, we demonstrated that conduction velocity increased over 21 days, yet connexin 43 expression did not appear to be more localized by 21 days. Studies have shown that increases in conduction velocity may not be solely connexin 43 dependent. Cardiac conduction can be determined by several factors including membrane excitability, intercellular coupling, as well as the size, shape and orientation of the cardiomyocytes (46, 47). de Boer et al. utilized a geometrically defined culture of immature cardiomyocytes and demonstrated that β-adrenergic induced increases in conduction velocity were due to changes in the intrinsic excitability of the cardiomyocytes and not alterations in gap junctional coupling. In adult mice with induced deletion of connexin 43, a 50% decrease in connexin 43 protein did not have any impact on conduction velocity (48). In a similar study, a 50% decrease in Scn5a, a gene that encodes sodium channel Na_v_1.5, demonstrated reduction in conduction velocity (49). To examine this phenomenon at the cellular level strands of neonatal cardiac myocytes with a 43% reduction in connexin 43 levels demonstrated no significant decrease in conduction velocity, but did demonstrate a significant increase in the action potential upstroke suggesting an increase in sodium channels. The data presented in these studies suggest that an increase in membrane excitability, plays an important role in conduction velocity. While not directly explored here, it is possible that connexin 43 localization was not the main driver for the increase in conduction velocity and that increases in membrane excitability and alignment have a role.

However, it is still important to note that many of these differences in alpha-actinin and connexin 43 organization and localization can be attributed to the immature phenotype of hPS-CM (50). This suggests that further steps will be necessary for enhanced maturation of connexin 43 localization and sarcomere organization of hPS-CM even given a substrate that promotes cell alignment. Others have shown that extended culture, as well incorporating electrical and mechanical stimulation can provide the cues necessary to impart maturation leading to improvements in cardiomyocytes functionality (50–53).

These findings suggest that hPS-CM are capable of attaching to fibrin microthreads, however a collagen IV protein coating improves hPS-CM attachment to fibrin microthreads. Additionally, hPS-CM were able to contract and align to the microthreads over 21 days in culture. By day 14 cell seeded microthreads were contracting at a frequency approaching that of a human heart and were producing strains nearing those produced by human myocardium. One of the limiting factors regenerative therapies for cardiac disease face is the inability to efficiently deliver cells to the area of damage. Many studies transplanting hPS-CM to diseased cardiac tissue have done so using a cardiac patch or cell sheet in order to improve cell retention (13, 54). However, these cell delivery methods have limited success due to the formation of collagen interfaces between the host and graft tissue, which limits the ability for graft cells to migrate into host tissue, thus limiting the potential for functional and electrical integration. This cell seeded microthread platform could influence cardiac cell therapy by utilizing the microthread as a delivery method by attaching a suture needle to the microthreads, as had been done previously (20, 55, 56). Doing so would provide a contractile fiber of cardiomyocytes that are able to be directly delivered to the damaged cardiac tissue to aid in regeneration of function in infarcted ventricular myocardium.

## Author Contributions

KH designed the study, collected, analyzed and interpreted data, and wrote the manuscript. ML provided the hPS-CM and contributed to study design, data interpretation, and manuscript editing. GG contributed to study design, data interpretation, manuscript writing, and provided financial support. All authors read and approved the final manuscript.

## Conflict of Interest Statement

The authors declare that the research was conducted in the absence of any commercial or financial relationships that could be construed as a potential conflict of interest.
