# Supplementary figures and images for: Development of a Contractile Cardiac Fiber From Pluripotent Stem Cell Derived Cardiomyocytes
Source: Front Cardiovasc Med. 2018 Jun 11;5:52. doi: 10.3389/fcvm.2018.00052 (PMC6004416; doi:10.3389/fcvm.2018.00052)

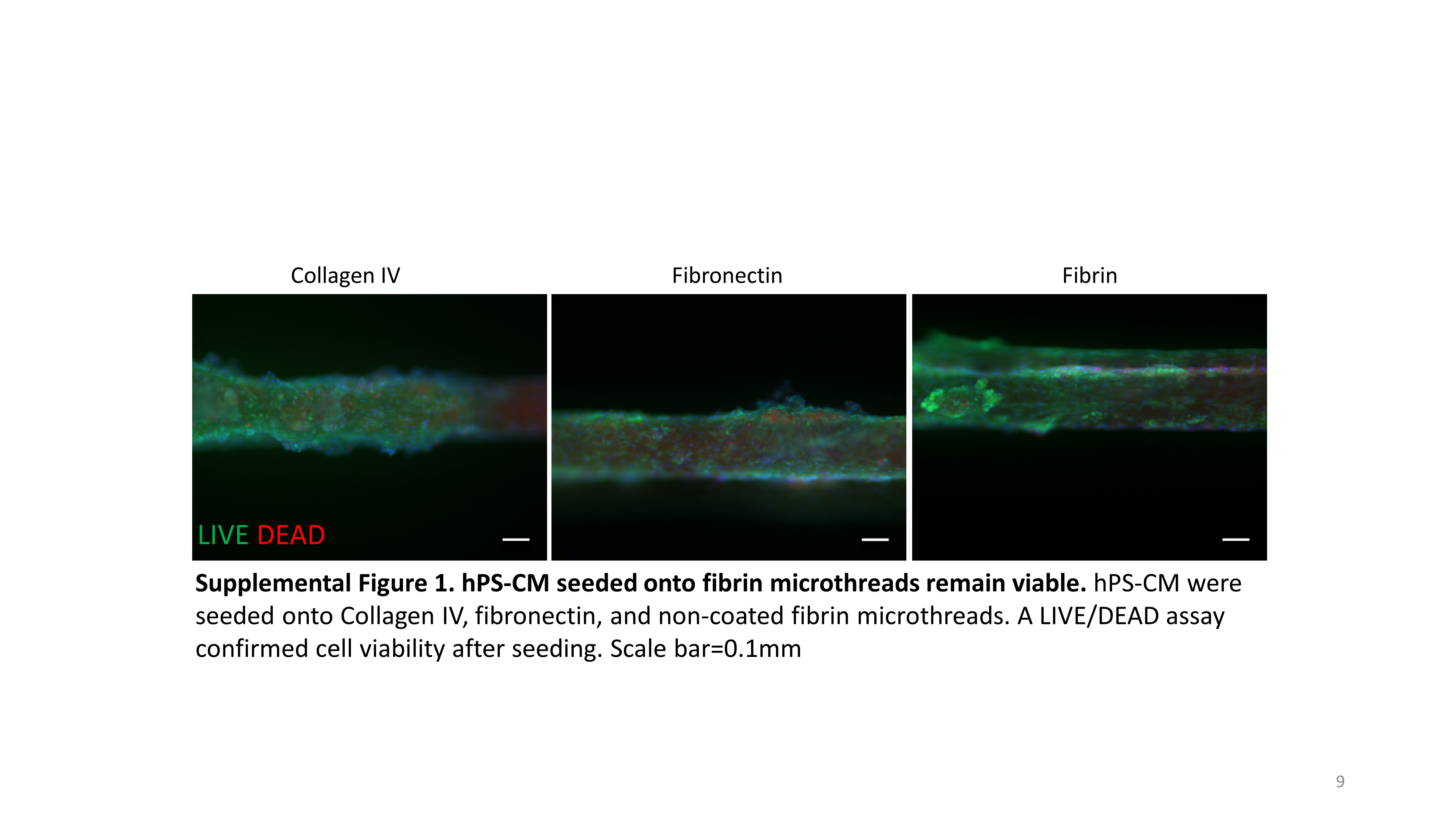

Supplement: Supplementary file 1 [file Image1.TIF]

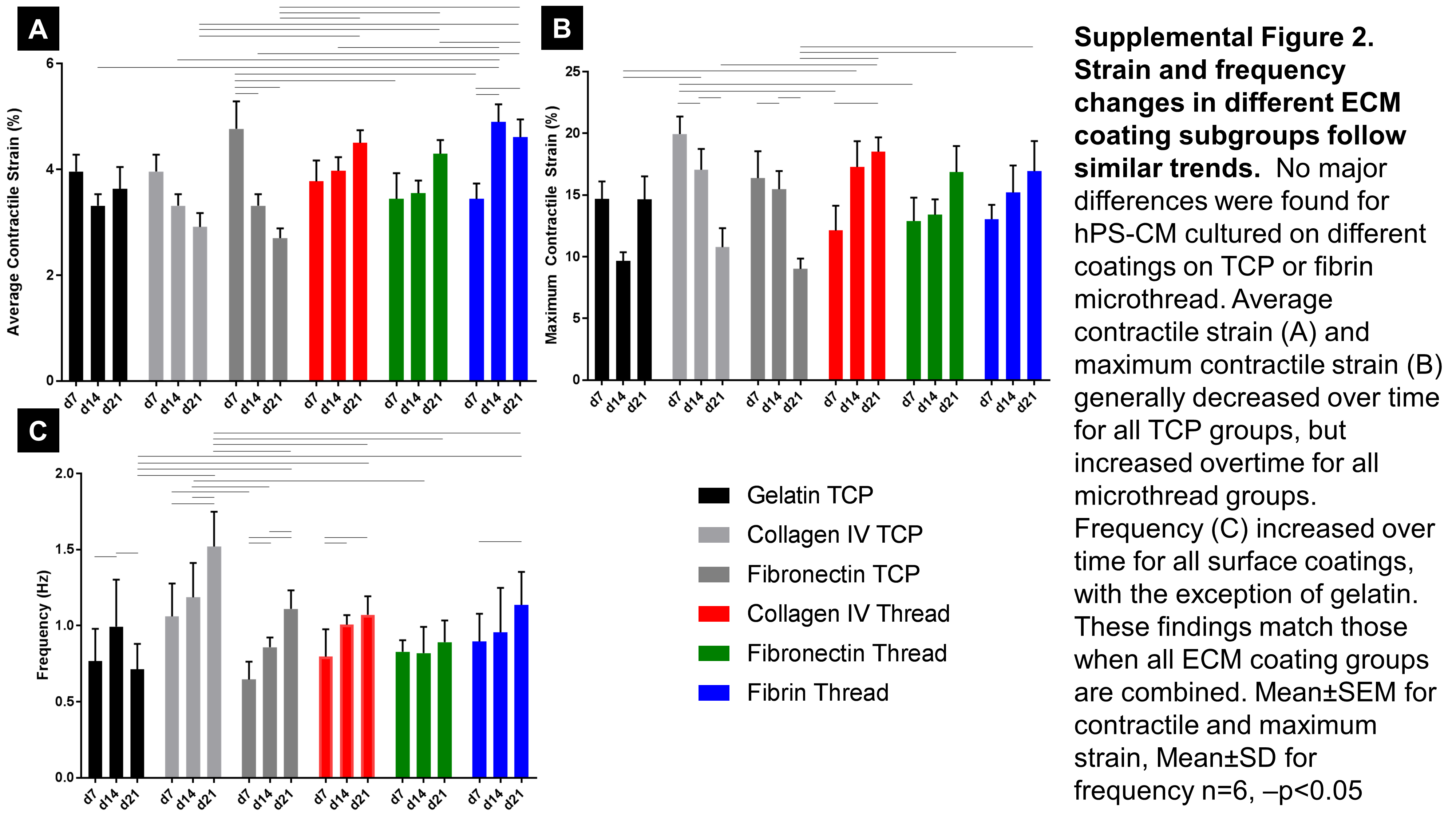

Supplement: Supplementary file 2 [file Image2.TIF]

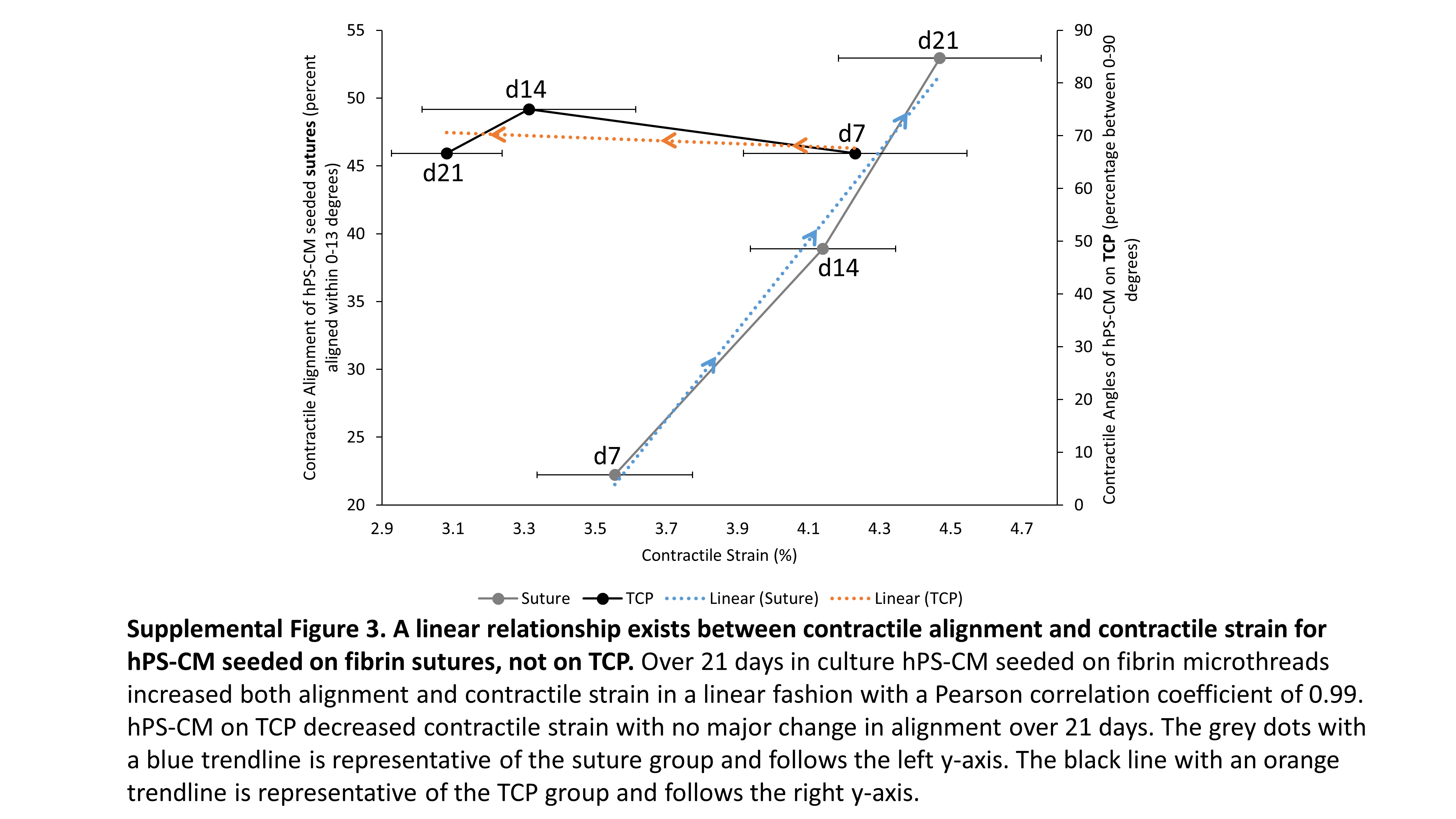

Supplement: Supplementary file 3 [file Image3.TIF]

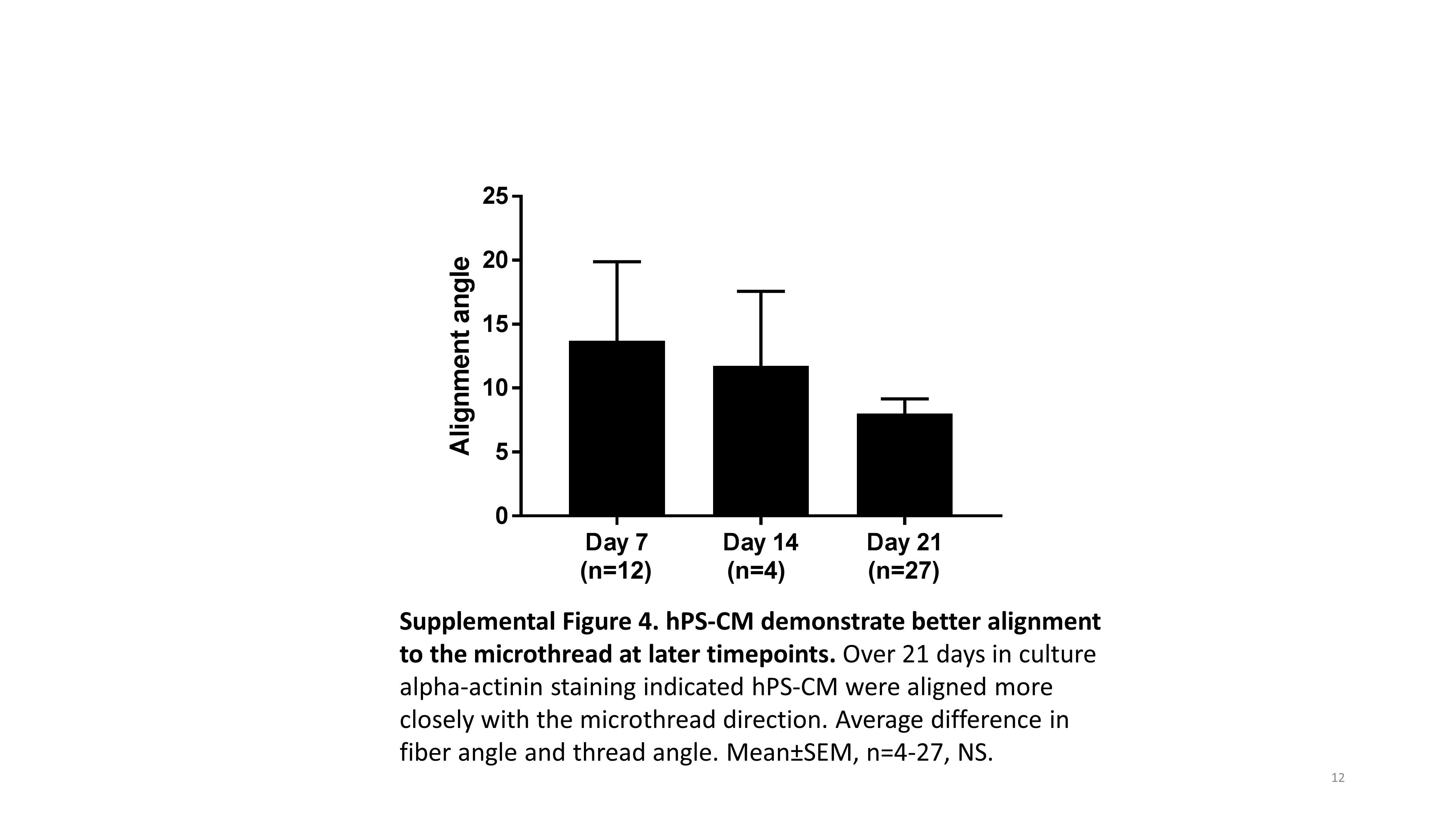

Supplement: Supplementary file 4 [file Image4.TIF]
